# Natural Products for Treatment of Alzheimer's Disease and Related Diseases: Understanding their Mechanism of Action

**DOI:** 10.2174/1570159x11311040001

**Published:** 2013-07

**Authors:** Rong Shao, Jianbo Xiao

Alzheimer’s disease (AD) is a progressive neurodegenerative disorder associated with memory impairment and cognitive deficit, which is characterized with low levels of acetylcholine, a neurotransmitter, in the brain of the patients. Histopathological hallmarks of AD include deposition of β-amyloid (Aβ) plaques and formation of neurofibrillary tangles. According to the cholinergic hypothesis, the acetylcholinesterase (AChE) catalyzes the hydrolysis of acetylcholine and the inhibition of AChE improves the levels of acetylcholine in the brain, enhancing cholinergic functions in patients. AChE inhibitors (AChEi) can alleviate the symptoms of AD, even though they do not halt or reverse the disease progress. Currently, most of the drugs approved by the US-FDA for the treatment of AD are AChEi. For example, galanthamine is a natural alkaloid obtained from Galanthus spp. Huperzine A, an alkaloid found in Huperzia spp., is an AChEi commercialized as a dietary supplement for memory support, used to treat AD symptoms in China. Extent reports on pathology of AD have made it important to discovery regarding mechanism of disease and potential therapeutic targets. This issue focused on the medicinal chemistry aspects of new natural products used for treatment of AD including the mechanism, the molecular aspects, as well as the new strategy of using natural products for AD.

The research on discovering new inhibitors of AChE has been focused on the compounds of both synthetic and natural origins. Murray *et al*. summarized natural AChE inhibitors from plants and their contribution to AD therapy, especially those published in the period from 2006 to 2012. More than 200 naturally-occurring compounds from plants were listed as potential new AChE inhibitors. Orhan specifically focused on the compounds with AChE inhibitory potential from natural sources including plants, animals, and microorganisms along with a brief summary of the conventional AChE-inhibiting natural compounds already in use.

Ansari and Khodagholi comprehensively reviewed the molecular mechanism aspects of natural products as promising drug candidates for the treatment of AD. They focused on some natural products with potential neuroprotective properties against Aβ with respect to their mechanism of action. Most of these compounds have remarkable antioxidant properties and act as free radical scavengers. Some of these compounds improve cell survival and enhance cognition by directly affecting amyloidogenesis and programmed cell death pathways. Ansari and Khodagholi also concluded that although neuroprotective compounds from natural sources are attractive therapeutic alternatives fro AD, poor bioavailability and low clinical efficacy are the major problems. Using novel pharmaceutical technologies and medicinal chemistry to look for novel formulations or to design new compounds based on natural templates are new strategies of using natural products for AD.

Due to its multi-faceted pharmacology, natural curcumin have been used for the treatment or prevention of neurodegenerative diseases such as AD, Parkinson’s disease (PD) and brain tumors. Lee *et al*. fully summarized the neuropharmacology and neuroscience of curcumin. The therapeutic benefits of curcumin for AD and PD appear multifactorial *via *regulation of transcription factors, cytokines, redox potential and enzymes associated with NFκB activity. Lee *et al*. discussed the pharmacology of curcumin and provide new perspectives on its therapeutic potential. They believe a multi-targeted and pleotropic therapy exhibits higher success in the treatment of AD and PD. 

Tanaka *et al*. carried out a systematic review of the studies that have analyzed the effect of *Ginkgo biloba *extract on PD. They gave two hypotheses for the positive effect of *G. biloba *extract on PD, namely the reduction or inhibition of monoamine-oxidase activity and the neuroprotective effect against 6-hydroxydopamine, 1-methyl-4-phenyl-1,2,3,6-tetrahydropyridine and MPP^+^ toxins.

